# Selective dendritic susceptibility to bioenergetic, excitotoxic and redox perturbations in cortical neurons^☆^

**DOI:** 10.1016/j.bbamcr.2014.12.021

**Published:** 2015-09

**Authors:** Philip Hasel, Sean Mckay, Jing Qiu, Giles E. Hardingham

**Affiliations:** Centre for Integrative Physiology, University of Edinburgh, Edinburgh EH8 9XD, UK

**Keywords:** Antioxidant defences, Oxidative stress, Excitotoxicity, Mitochondria, Calcium signalling, Bioenergetics

## Abstract

Neurodegenerative and neurological disorders are often characterised by pathological changes to dendrites, in advance of neuronal death. Oxidative stress, energy deficits and excitotoxicity are implicated in many such disorders, suggesting a potential vulnerability of dendrites to these situations. Here we have studied dendritic vs. somatic responses of primary cortical neurons to these types of challenges in real-time.

Using a genetically encoded indicator of intracellular redox potential (Grx1-roGFP2) we found that, compared to the soma, dendritic regions exhibited more dramatic fluctuations in redox potential in response to sub-lethal ROS exposure, and existed in a basally more oxidised state. We also studied the responses of dendritic and somatic regions to excitotoxic NMDA receptor activity. Both dendritic and somatic regions experienced similar increases in cytoplasmic Ca^2+^. Interestingly, while mitochondrial Ca^2+^ uptake and initial mitochondrial depolarisation were similar in both regions, secondary delayed mitochondrial depolarisation was far weaker in dendrites, potentially as a result of less NADH depletion. Despite this, ATP levels were found to fall faster in dendritic regions. Finally we studied the responses of dendritic and somatic regions to energetically demanding action potential burst activity. Burst activity triggered PDH dephosphorylation, increases in oxygen consumption and cellular NADH:NAD ratio. Compared to somatic regions, dendritic regions exhibited a smaller degree of mitochondrial Ca^2+^ uptake, lower fold-induction of NADH and larger reduction in ATP levels. Collectively, these data reveal that dendritic regions of primary neurons are vulnerable to greater energetic and redox fluctuations than the cell body, which may contribute to disease-associated dendritic damage. This article is part of a Special Issue entitled: 13th European Symposium on Calcium.

## Highlights

• Dendrites exhibit a greater shift in redox potential than the soma, following an oxidative insult.
• Dendritic mitochondria depolarise less than somatic ones during excitotoxicity.
• Nevertheless ATP falls faster in dendritic regions during excitotoxicity.
• Energetically demanding AP bursting induces adaptive metabolic responses.
• These responses are weaker in dendrites, and ATP levels are suppressed more strongly.

## 1 Introduction

In many chronic and acute conditions of the brain involving neuronal dysfunction there is substantial evidence that the effects of a diverse array of disease-causing events, agents and mutations at least partly converge on a common set of consequences centred on excitotoxicity, energy imbalance, oxidative stress and mitochondrial dysfunction [55,70,71,74,91].

Excitotoxicity is caused by the global or local accumulation of glutamate, leading to inappropriate activity of the NMDA subtype of glutamate receptor (NMDAR) which mediates Ca^2+^-dependent cell death and dysfunction [17,61]. This ‘excitotoxicity’ is a major contributor to neuronal loss and dysfunction in acute neurological disorders including stroke and traumatic brain injury [60]. However, more local, progressive excitotoxicity is also implicated in the pathogenesis of neurodegenerative disease [62,80]. For example, synaptic loss in Alzheimer's disease (AD) models induced by oligomeric Aβ is thought to proceed at least in part via a local NMDAR-dependent excitotoxic mechanism [53,66]. Huntington's disease is also a disorder associated with aberrant NMDAR activity and excitotoxicity [29], in part due to an elevation of extrasynaptic NMDAR activity which promotes neuronal dysfunction as well as preventing non-toxic mHtt inclusion formation [72,77].

The principle cause of excitotoxicity in stroke is a loss of bioenergetic homeostasis, leading to dysfunctional glutamate uptake mechanisms and accumulation of extracellular glutamate. Furthermore there is considerable evidence that energetic and metabolic perturbations occur in chronic neurodegenerative diseases, not just acute disorders such as stroke or traumatic brain injury [58]. Central to energy homeostasis, of course, is the requirement that functional mitochondria are able to respond to the changing demands of a neuron (for example during periods of strong synaptic activity). Dysfunctional mitochondria are a hall-mark of many chronic and acute neurological disorders, which can be triggered by both excitotoxic and non-excitotoxic mechanisms. An example of the latter is oxidative stress, which is not only triggered by excitotoxicity and a variety of disease-causing agents and mutations, but can also be further exacerbated by mitochondrial dysfunction.

Given that neurons are highly polarised cells with many spatially and biochemically distinct regions, it is perhaps not surprising that these regions are differentially affected under pathological conditions. For example, there is good evidence that changes in dendritic and axonal morphology and properties take place in neurodegenerative disease in advance of neuronal loss [65]. In AD and mouse models of AD, dystrophic neurites and spine alterations correlate with proximity to amyloid plaques [35,87]. Moreover, in animal models of AD dendritic alterations have been observed in advance of plaque formation [84] and recent evidence suggests that structural dendritic deficits in an AD model are functionally linked to hyperexcitability [85]. α-Synuclein inclusions promote mitochondrial oxidative stress in dopaminergic neurons that is higher in dendritic regions than in the soma, suggestive of dendritic vulnerability in Parkinson's disease [25]. Even normal ageing is associated with a reduction in dendritic complexity and spine numbers [23]. Acute excitotoxicity such as exposure to NMDA or oxygen–glucose deprivation also triggers early dendritic alterations in the form of neuritic beads [36], although their role (protective or pathological) is not well understood.

Despite this, we have an incomplete picture of how dendritic vs. somatic regions respond in real-time to metabolic, oxidative and excitotoxic stress. This knowledge could help understand the types of insult that trigger selective vulnerability to more peripheral regions of a neuron, as well as indicate whether classical neuroprotective strategies are appropriate for preventing more localised dendritic or synaptic damage. Key to gaining a better understanding of real-time responses of neurons to various forms of stress has been the development of an expanding array of genetically encoded indicators designed to report on a variety of metabolic intermediates, second messenger levels, and signal pathway activity. Particularly important for studying dynamic neuronal responses to excitotoxic, metabolic and oxidative stress include mitochondrially targeted Ca^2+^ indicators, probes for NADH:NAD ratio, ATP levels, AMPK activity and cellular redox state (GSH:GSSG ratio). These indicators can be usefully employed in parallel with more classical imaging techniques, such as charged dye reporting of mitochondrial membrane potential, and NAD(P)H autofluorescence measurements.

Here we have employed these existing probes, plus a newly targeted version of GCaMP2, to compare dendritic vs. somatic responses of cortical neurons to excitotoxic insults (lethal and sub-lethal), a sub-lethal oxidative challenge, and finally the metabolic demands of intense synaptic activity. As well as illustrating the utility of these probes, we found that compared to the soma, dendrites were more susceptible to strong fluctuations in redox state and ATP levels. However, dendritic mitochondria appeared more resistant to catastrophic depolarisation during exposure to excitotoxic insults.

## 2 Results

### 2.1 Analysis of subcellular redox potential changes following H_2_O_2_ exposure

**Fig. 1:**
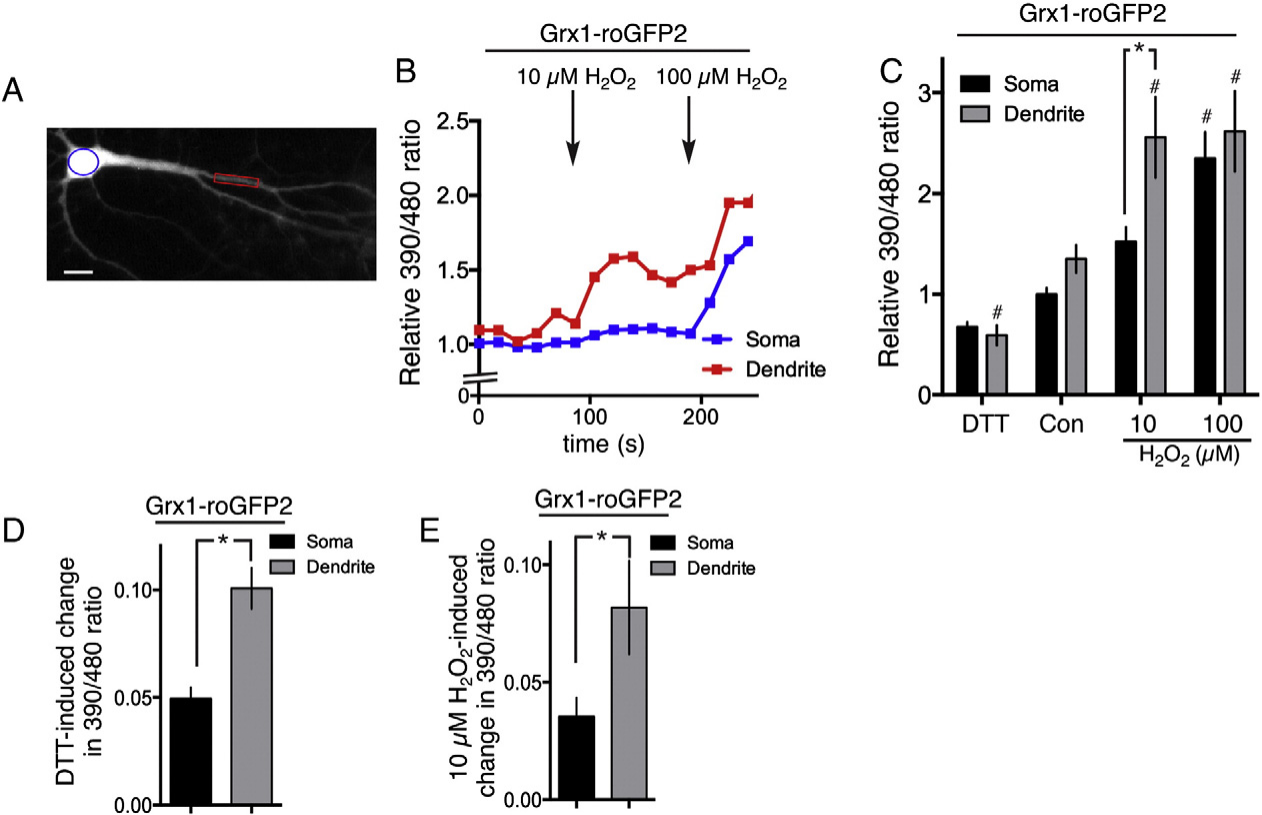
Dendritic regions are subject to greater fluctuations in the GSH redox potential after exposure to sub-toxic H_2_O_2_. A) Example of a Grx1-roGFP2 expressing neuron, with the soma (blue) and dendrite (red highlighted). Scale bar = 10 μm. B, C) Grx1-roGFP2 expressing neurons were treated (DTT at 10 mM) as indicated and the fluorescence ratio (ex: 387 ± 5:494 ± 10, em: 530 ± 10) calculated, and normalised to the pre-treatment (Con) somatic ratio of that cell. *p < 0.05 two way ANOVA plus Bonferroni's post-hoc test (n = 9–23). ^#^p < 0.05 comparing that condition to untreated (control) condition for that particular subcellular region (two way ANOVA plus Dunnett's post-hoc test (n = 9–23)). D, E) The change in Grx1-roGFP2 390/480 ratio induced by either DTT (D) or 10 μM H2O2 (E) is shown. *p < 0.05 Student t-test (n = 14 (D), n = 9 (E)).

We first decided to compare dendritic vs. somatic responses to an oxidative challenge. Classical probes are based on non-fluorescent dyes which, upon oxidation, become fluorescent. However, this is a one-way reaction and so does not indicate recovery of redox status. To visualise the redox potential of the cell directly, we transfected neurons with a vector encoding the Grx1-roGFP2 genetically encoded reporter of the glutathione redox potential [42] (Fig. 1A). The 390/480 nm excitation ratio of Grx1-roGFP2 is highly sensitive to small fluctuations in redox potential. Its dynamic range (−320 to −240 mV [42]) means that small changes in the degree of GSH oxidation lead to large changes in Grx1-roGFP2 signal [42] and so it is ideal for measuring cellular responses to sub-toxic oxidative challenges. We found that low doses of H_2_O_2_ produced strong fluctuations in the probe signal which were frequently saturated at above 25 μM H_2_O_2_. Using a H_2_O_2_ dose well within the dynamic range (10 μM H_2_O_2_) we monitored the fluctuation in glutathione redox potential in somatic vs. dendritic regions, normalised to the maximal response elicited by a high dose of H_2_O_2_ (100 μM). We found that dendritic regions exhibited stronger perturbations to the glutathione redox potential, compared to the soma of that same cell. Fig. 1B shows an example recording from the dendritic and somatic regions of a single cell, and Fig. 1C shows the full data set, and Fig. 1E shows the 10 μM H_2_O_2_-induced change in both regions. We also observed that dendritic Grx1-roGFP2 ratios tended to be higher in dendritic regions compared to their soma, suggesting an elevated basal oxidation state. To test this, in a set of cells we treated them with DTT to provide highly reducing conditions in order to minimize the 390/480 nm excitation ratio. We found that the effect of DTT was greater in dendritic regions than in somatic regions, suggesting that dendrites are indeed in an elevated basal oxidation state.

### 2.2 Analysis of subcellular metabolic and mitochondrial changes during excitotoxicity

**Fig. 2:**
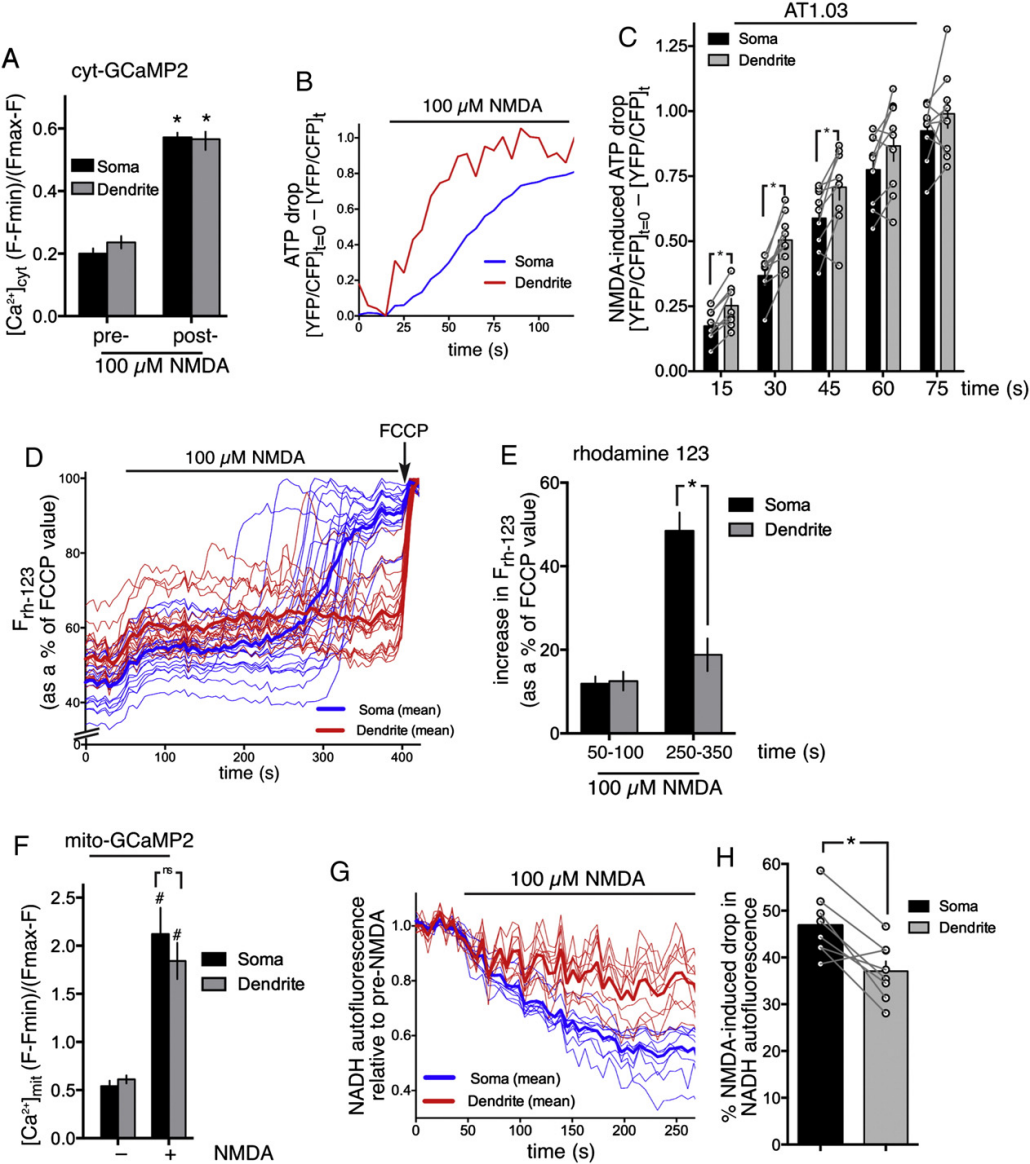
During excitotoxicity dendrites suffer more rapid ATP loss but less mitochondrial dysfunction. A) Ca^2+^ imaging of neurons expressing Cyt–GCaMP2 before and after treatment with NMDA. Average Ca^2+^ concentration over the 60 s post NMDA treatment in somata and dendrites is shown. *p < 0.05 paired t-test compared to pre-stimulation level (n = 28). B, C) Neurons expressing AT1.03 were treated with NMDA as indicated and the YFP:CFP FRET ratio was calculated (ex: 427 ± 5 nm; em: 427 ± 15: 542 ± 13). The change in FRET ratio post-NMDA treatment is shown (value at t = 0 minus value at t), with an increasing value indicating a decreasing FRET ratio (meaning a reduction in ATP). (B) shows example trace, (C) shows quantitation. *p < 0.05 two way ANOVA plus Bonferroni's post-hoc test (n = 8). D, E) Neurons loaded with rhodamine-123 were treated with NMDA and fluorescence measured in somatic and dendritic regions, expressed as a percentage of the maximum obtained upon addition of FCCP. (D) shows a single experiment tracking 20 somatic and 20 dendritic regions (thin lines) and their means (thick lines). (E) shows the maximum depolarisation within two time windows that correspond to primary (50–100 s post-NMDA) and secondary (250–350 s post-NMDA) phases of somatic depolarisation. *p < 0.05 t-test (80 somatic and dendritic regions within n = 4 independent experiments). F) Ca^2+^ imaging of neurons expressing mito-GCaMP2 before and after treatment with NMDA. Average Ca^2+^ concentration over the 60 s post-NMDA treatment in somata and dendrites is shown. ^#^p < 0.05 paired t-test compared to pre-stimulation level (n = 12). G, H) NAD(P)H autofluorescence (ex: 387 ± 5, em: 447 ± 30) was measured before and after NMDA treatment. (G) shows an example of a single experiment tracking 10 somatic and 10 dendritic regions (thin lines) and their means (thick lines). (H) shows the % drop in NAD(P)H autofluorescence (recorded until autofluorescence plateaued at ~ 300 s post NMDA). *p < 0.05 t-test (80 somatic and dendritic regions within n = 8 independent experiments).

We next investigated somatic and dendritic responses to excitotoxic Ca^2+^ influx through the NMDA receptor (NMDAR). We first studied the consequences of excitotoxic NMDAR activity for cellular ATP levels in dendritic and somatic regions. We used the genetically encoded ATP FRET probe AT1.03 [52], which shows a YFP/CFP emission ratio of around 1.6 in resting neurons, and which falls to around 0.6 upon depletion of cellular ATP achieved by treatment with the mitochondrial uncoupler FCCP (data not shown). Application of NMDA (100 μM) elicited similar increases in cytoplasmic Ca^2+^, measured using the cytoplasmic indicator GCaMP2 (Fig. 2A). However, we found that the rate of loss of ATP, as measured using AT1.03, was more rapid in dendritic regions than in the soma over early time points (Fig. 2B, C).

Loss of mitochondrial membrane potential (Ψm) is an early event in excitotoxicity, initially triggered by mitochondrial Ca^2+^ uptake [1,82], but later irreversible and Ca^2+^-independent, involving PARP-mediated depletion of NADH [1]. We therefore decided to investigate whether the mitochondria in these different regions respond differently to excitotoxic Ca^2+^ influx. Using the Ψm probe rhodamine-123 (Rh123), we studied the loss of Ψm in response to bath application of NMDA. Rh123 partitions into the polarised mitochondria of neurons where its accumulation causes self-quenching. When the mitochondria depolarise during exposure to excitotoxic insults, Rh123 moves increasingly into the cytoplasm, whereupon it de-quenches and its fluorescent signal increases [57,86]. The fluorescence signal is normalised cell-to-cell and region-to-region by calculating the maximal fluorescence at the end of the experiment upon complete mitochondrial depolarisation (achieved by FCCP treatment). We observed an initial slow modest depolarisation in the soma of neurons, followed by a secondary, more dramatic loss of Ψm (Fig. 2D, E). This has been described by others and may involve cellular NADH loss and permeability transition [1]. In dendritic regions however, while the initial slow modest depolarisation was also observed, the secondary loss of Ψm was far less dramatic, and in some cases missing entirely (Fig. 2D, E).

To investigate a potential reason for this, we measured Ca^2+^ uptake into the mitochondria using matrix-targeted mito-GCaMP2, and found no significant differences between uptake in dendritic vs. somatic regions (Fig. 2F). The fact that matrix Ca^2+^ rises to similar levels in the somatic and dendritic mitochondria is consistent with the similar levels of initial mitochondrial depolarisation, but begs the question as to why secondary depolarisation is much stronger in somatic regions. Since NADH depletion is an important mediator of the secondary component of mitochondrial depolarisation [1], differences in NADH depletion offer another explanation. We studied NAD(P)H autofluorescence which, since NADH is in excess over NADPH, primarily reports NADH levels. As with previous studies, we observed that NADH autofluorescence was concentrated in the mitochondria throughout the neuron. We found that the proportional drop in NADH in somatic regions was significantly greater than in dendritic regions (Fig. 2G, H), offering a potential explanation for the more dramatic mitochondrial depolarisation observed in the soma. Collectively, these data employing a range of imaging approaches reveal distinct differences in the response of the soma and dendrites to relatively uniform levels of NMDAR-dependent Ca^2+^ influx.

### 2.3 Analysis of adaptive metabolic responses to network-wide action potential burst activity

**Fig. 3:**
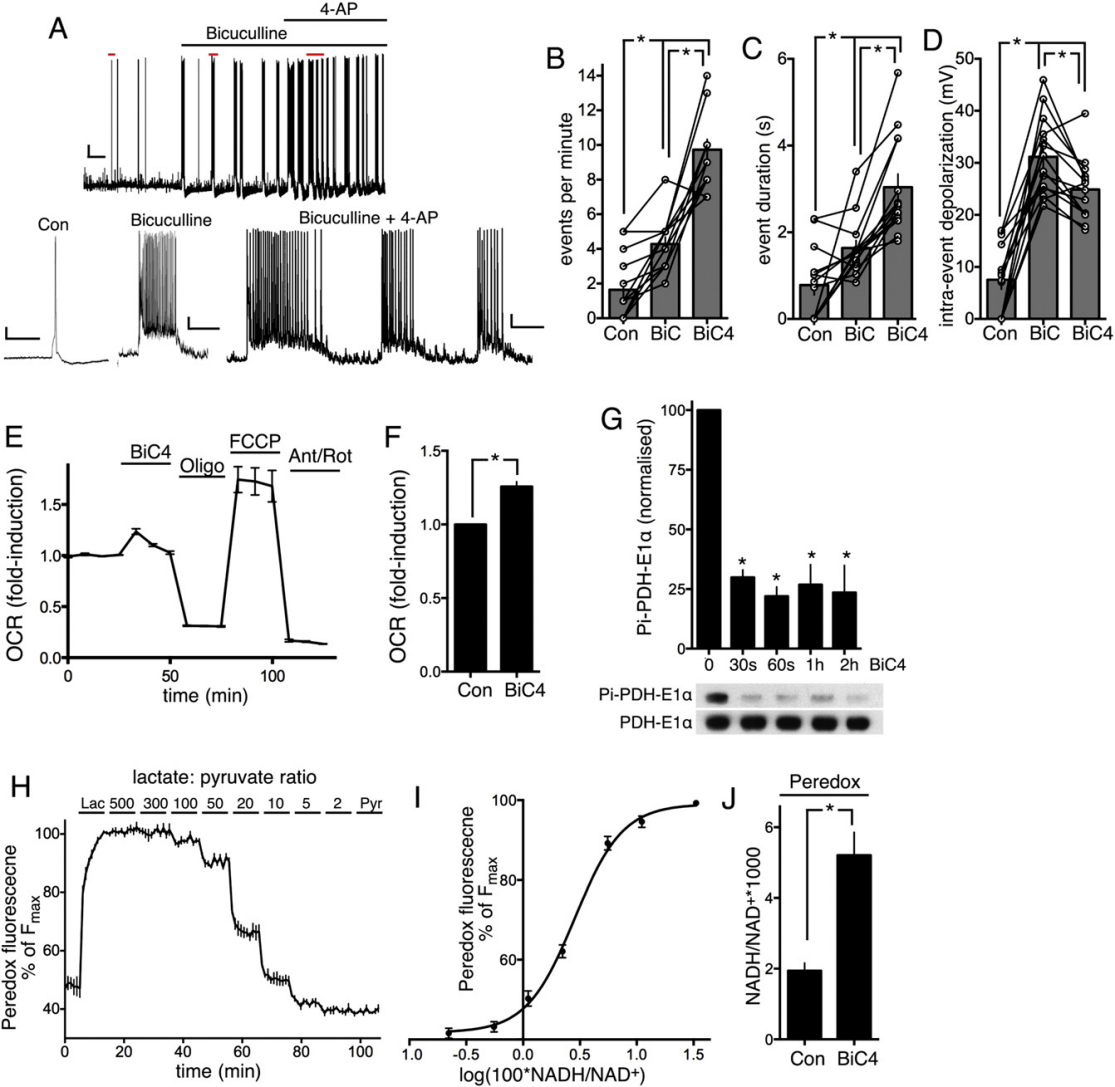
Network disinhibition triggers strong AP bursting and increased metabolic activity. A) Example whole-cell current clamp recording of a neuron treated as indicated with bicuculline (50 μM) ± 4-aminopyridine (250 μM). Regions marked in red in the upper picture are expanded in the lower pictures. Scale bars: 10 mV/10s (upper); 10 mV/50 ms (lower left); and 10 mV/1 s (lower middle and left). B–D) Effect of bicuculline (BiC) or BiC/4-AP (BiC4) on neuronal activity. The frequency of episodes of firing activity (B), their duration (C) and the average amount of intra-episode depolarisation (D) are shown. *p < 0.05 ANOVA plus Bonferroni's post-hoc test (n = 14). E, F) Example experiment showing an increase in oxygen consumption rate (OCR) by BiC/4-AP treatment (BiC4). Subsequent treatment with oligomycin (1 μM) illustrates that OCR is driven primarily by mitochondrial ATP production. Treatment with the uncoupler FCCP (0.125 μM) uncovers the maximal OCR rate, and the OCR after treatment with antimycin (2 μM) plus rotenone (2 μM) (Ant/Rot) represents non-mitochondrial oxygen consumption. (F) shows quantitation of BiC/4-AP-induced OCR. *p < 0.05 t-test (n = 17). G) Western analysis of PDH dephosphorylation in response to BiC/4-AP-induced burst activity. *p < 0.05 t-test (n = 3). H) Example trace showing the fluorescence of Peredox (ex: 387 ± 5, em: 530 ± 10, normalised to its mCherry tag ex: 575 ± 12, em: 628 ± 24) in neurons exposed to different ratios of lactate:pyruvate, which alters the cellular NADH:NAD ratio. I) From the calibration data in (H), plus knowledge of the equilibrium constant k = 1.11 ∗ 10^− 4^ of lactate dehydrogenase (to convert lactate:pyruvate to NADH:NAD), the relationship of Peredox fluorescence to NADH:NAD is calculated. See methods for further details. J) Effect of BiC/4-AP (BiC4)-induced burst activity on cellular NADH:NAD ratio. *p < 0.05 t-test (n = 19).

While Ca^2+^ influx through the bath application of high concentrations of NMDA triggers excitotoxic death, physiological patterns of Ca^2+^ influx, such as those triggered by synaptic activity or action potential bursting, are well-tolerated and indeed can trigger long-lasting neuroprotective adaptive responses [9]. Nevertheless, electrical activity is energetically and metabolically demanding, particularly at the synapses [50], and is known to trigger rapid adaptive responses to meet this increased demand [44,50]. Against this context we wanted to know whether burst activity triggered different responses in dendrites vs. the soma with regard to energetic and metabolic homeostasis. Under basal conditions, firing activity is relatively variable cell-to-cell, with ‘events’ ranging from single action potentials (APs) to short bursts. Activity can be enhanced by network disinhibition achieved by treatment with the GABA_A_ receptor antagonist bicuculline [48]. This results in an increase in AP burst duration and frequency (Fig. 3A–C), while at the same time rendering responses far more homogeneous cell-to-cell (since the activity is synchronized across the network). In addition, the average degree of depolarisation during each event is substantially increased (Fig. 3D). In order to enhance the metabolic demands on the cell still further, we co-treated neurons with bicuculline along with the weak K^+^ channel blocker 4-aminopyridine (4-AP) [48], which enhances both burst frequency and duration (Fig. 3A–C), albeit with a slight reduction in average intra-event depolarisation (Fig. 3D).

We observed clear evidence of increased metabolic activity to counter the increased energy demand of firing activity triggered by bicuculline/4-AP. Oxygen consumption rate (measured by a Seahorse bioanalyzer) was elevated, indicative of increased oxidative phosphorylation (Fig. 3E, F). Moreover we observed activating dephosphorylation (on serine 293) of pyruvate dehydrogenase (Fig. 3G), which increases flux through the TCA cycle [83] and may contribute with Aralar activation to boost oxidative phosphorylation. To determine whether this resulted in increased NADH production, we employed the genetically encoded indicator of cellular NADH:NAD ratio Peredox [51]. We used the nuclear-targeted form of Peredox because (as previously reported) expression of the cytoplasmic version results in lysosomal inclusions in neurons [51]. We first validated the probe in our hands using varying ratios of lactate and pyruvate to alter the cellular NADH:NAD ratio (Fig. 3H, I). We then studied the impact of BiC/4-AP treatment and observed a strong increase in the Peredox-reported NADH:NAD ratio, consistent with increased NADH production (Fig. 3J).

### 2.4 Analysis of subcellular metabolic responses during action potential burst activity

**Fig. 4:**
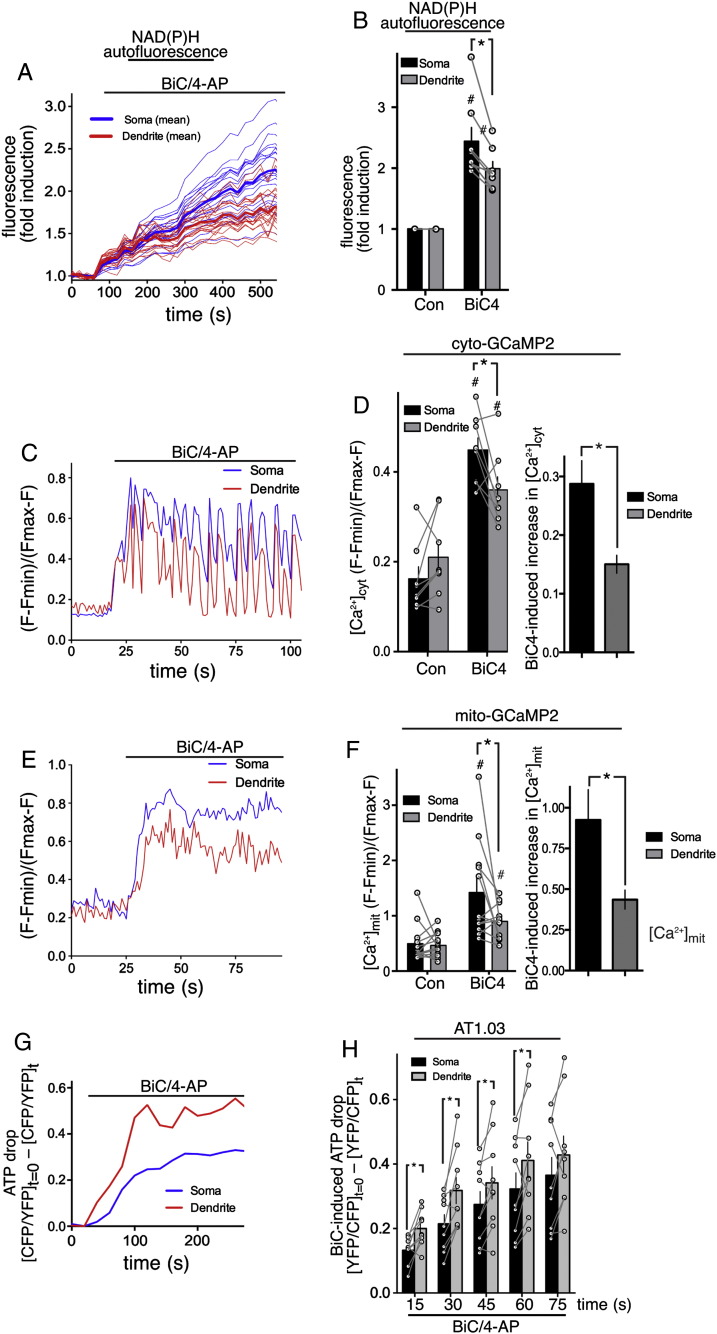
In response to AP bursting dendritic regions experience lower Ca^2+^ levels, exhibit weaker NADH production, and suffer higher ATP losses. A, B) NAD(P)H autofluorescence was measured before and after BiC/4-AP (BiC4) treatment. (A) shows an example of a single experiment tracking 20 somatic and 20 dendritic regions (thin lines) and their means (thick lines). (B) shows the fold increase in NAD(P)H autofluorescence 10–16 min post-BiC/4-AP (BiC4). *p < 0.05 t-test (160 somatic and dendritic regions within n = 8 independent experiments). C–F) Ca^2+^ imaging of neurons expressing cyto-GCaMP2 (C, D) or mito-GCaMP2 (E, F) before and after treatment with BiC/4-AP. (C) and (E) show example traces depicting the Ca^2+^ levels within a single cell (dendrite vs. mitochondria). (D, left) and (F, left) show the average Ca^2+^ concentration over the 60 s pre- and post-stimulation in somata and dendrites. (D, right) and (F, right) show the *difference* between pre- and post-BiC/4-AP stimulation Ca^2+^ levels. ^#^p < 0.05 compared to pre-stimulation level; *p < 0.05 soma vs. dendrite (n = 12 mito-GCaMP2 and n = 8 cyto-GCaMP2 cells). G, H) Neurons expressing AT1.03 were treated with BiC/4-AP as indicated and the YFP:CFP FRET ratio calculated. The change in FRET ratio post-treatment is shown (value at t = 0 minus value at t), with an increasing value indicating a decreasing FRET ratio (meaning a reduction in ATP). (G) shows example trace, and (H) shows quantitation. *p < 0.05 two way ANOVA plus Bonferroni's post-hoc test (n = 7).

Since Peredox did not give any spatial information on NADH levels, we had to resort to imaging NAD(P)H autofluorescence to study somatic vs. dendritic responses. We observed increases in NADH autofluorescence in both somatic and dendritic areas, suggestive of enhanced flux through the TCA cycle in both regions. However, the fold increase in NADH levels was lower in dendritic regions than in somatic ones (Fig. 4A, B). Since Ca^2+^ influx and uptake into the mitochondria is likely to be a driver of enhanced NADH production, we investigated whether there were any differences in activity-dependent Ca^2+^ dynamics at somatic vs. dendritic locations. Using cytoplasmic GCaMP2 we observed that Ca^2+^ elevation in somatic regions was on average higher than in dendritic regions (Fig. 4C, D). In particular we observed that somatic Ca^2+^ levels remained high between bursts, while in dendritic regions they tended to fall down towards baseline levels (Fig. 4C). We have reported these differences in Ca^2+^ dynamics previously in rat hippocampal neurons which we attributed to more efficient Ca^2+^ clearance from dendritic regions [48]. As a likely consequence of these differences in cytoplasmic Ca^2+^ dynamics, levels of activity-dependent mitochondrial Ca^2+^ elevation were also lower in dendritic regions than in somatic regions (Fig. 4E, F). Finally we looked at ATP levels, to determine whether the adaptive responses of somatic vs. dendritic regions differed in their capacity to counter the energetic demands in their respective subcellular locations. We found that firing activity caused a depression of cellular ATP levels, smaller than those observed in response to excitotoxic insults but significant nonetheless. Of note, we found that the drop in ATP levels was more pronounced in dendritic regions than in the soma (Fig. 4G, H), suggestive of a greater imbalance between energy use and supply in dendrites during periods of intense activity.

## 3 Discussion

Here we have shown significant differences in how dendrites and soma respond to the same challenge, be it oxidative, excitotoxic or activity-dependent. This adds to a body of work reporting selective dendritic responses to these types of insult, but raises the question as to what the basis for these differences is.

### 3.1 Dendritic vs. somatic responses to ROS exposure

Oxidative stress occurs when there is an imbalance between the level of ROS and a cell's ability to neutralise them utilising their intrinsic antioxidant defences, while mild oxidative perturbations can trigger adaptive protective responses [10,11,45,47]. Our study revealed that exposure of cortical neurons to low levels of H_2_O_2_ triggered a greater shift in the GSH redox potential in dendrites compared to the soma of the same neuron. Several factors can influence how strongly the GSH redox potential is perturbed, including the rate of production of GSH, the reduction of oxidised GSSG back to GSH, and of course the oxidation of GSH by GSH peroxidases to neutralise H_2_O_2_. The stronger perturbation of the GSH:GSSG ratio in dendrites upon exposure to H_2_O_2_ could conceivably be due to dendritic vs. somatic differences in one or all of these factors. However, an additional consideration is the greater surface area: volume ratio of dendrites compared to somata, resulting in a higher amount of H_2_O_2_ flux into the cytoplasm relative to the volume of cytoplasm available to reduce it. It will also be of interest to know whether very prolonged exposure to H_2_O_2_ results in the GSH:GSSG ratios in both compartments becoming more similar. Deregulation of glutathione homeostasis and other antioxidant systems is implicated in the aetiology of several neurodegenerative disorders associated with dendritic pathologies, including: Alzheimer's disease, Huntington's disease, ALS Friedreich's ataxia, and Parkinson's disease [8,54,65], and activation of GSH pathway enzymes, particularly in astrocytes, is a potential therapeutic strategy for combating oxidative stress in the brain [39,41]. Moreover, deficits in the GSH system have been implicated in the pathophysiology of neuropsychiatric disorders, including schizophrenia, bipolar disorder and autistic spectrum disorder [24,30,31,33,38,43,59,69], associated with more subtle dendritic disturbances such as spine alterations. One would expect that any perturbations to the GSH system would disproportionately affect dendritic (and axonal) regions upon exposure to ROS, either generated endogenously or exogenously (e.g. by microglia).

### 3.2 Dendritic vs. somatic responses to excitotoxic Ca^2+^ influx

The neurotoxicity of sustained glutamate exposure [64], later termed excitotoxicity [78] is predominantly down to excessive Ca^2+^ influx through the NMDAR [16,18,19], and can kill human neurons as well as rodent ones [40]. The work of many laboratories have contributed to our understanding of how glutamate dyshomeostasis, ionic imbalance and abnormal NMDAR activity can contribute to excitotoxicity in a variety of acute and chronic disorders [5,14,21,28,29,56,62,76].

Mechanisms of excitotoxicity can differ depending on the intensity of insult [4,12], although other factors such as synaptic/extrasynaptic location and subunit composition also matter [46,88,89]. Acute excitotoxicity (as used in this current study) is associated with a rapid drop of ATP, loss of mitochondrial membrane potential, and delayed Ca^2+^ deregulation [1,26]. Collapse of the mitochondrial membrane potential following NMDAR activation requires Ca^2+^ influx and is prevented by inhibitors of the potential driven mitochondrial Ca^2+^ uniporter [57], a channel whose molecular identity and accessory factors have been recently uncovered [7,20,22]. However, events other than mitochondrial Ca^2+^ uptake are required for mitochondrial membrane potential collapse. Current models suggest that increased ROS (especially superoxide) production induced by mitochondrial Ca^2+^ uptake [26], and by cytoplasmically-activated ROS sources such as NADPH oxidase [13], combined with NO induced by nNOS activation leads to peroxynitrite-induced DNA damage, resulting in PARP-1 activation [26]. PARP-1 activation can then lead to the release of apoptotic factors from the mitochondria [90] as well as cellular NAD depletion and/or inhibition of glycolysis [3], leading to collapse of mitochondrial membrane potential due to a loss of the supply of reducing equivalents to the electron transport chain [1]. Our observations regarding the subcellular responses of somata vs. dendrites to excitotoxic insults suggest a disconnection between the severity of ATP depletion and the degree of mitochondrial depolarisation.

Following NMDA application, matrix Ca^2+^ levels rise equally in somata and dendrites (Fig. 2), and the immediate modest loss of mitochondrial membrane potential (likely a direct consequence of positively charged Ca^2+^ uptake), is also similar in both regions. However, while mitochondrial depolarisation and NADH depletion is more modest in dendritic regions, ATP loss is actually more rapid (Fig. 2). One possible explanation for the reduced NADH depletion is that PARP-1 activity is lower in dendritic regions, which is certainly a possibility since the current dogma states that PARP-1 activation is triggered by DNA damage in the nucleus. This could then explain why mitochondrial depolarisation is more modest in dendritic regions. However, it does not explain the rapid loss of ATP observed in dendrites. One possibility is that ATP utilization is much higher in dendritic regions, for example to maintain cytoplasmic Ca^2+^ concentrations at healthy levels in the face of high rates of Ca^2+^ influx. It is easy to envisage that dendritic regions have a higher ratio of NMDARs to cytoplasmic volume than somatic regions, meaning a greater demand on ATP-utilising plasma membrane and ER Ca^2+^ pumps. Regardless, it is clear that in both regions mitochondrial depolarisation is not the main trigger for ATP loss, given that the former occurs well after the latter. Accelerated ATP loss within dendrites may be a consequence of unsustainable demands on the Na^+^/K^+^ ATPase in dendrites due to Ca^2+^ and Na^+^ influx [37]. This increased ion flux also contributes to morphological changes to dendrites, such as dendritic beading [37,73] and spine loss [73].

### 3.3 Dendritic vs. somatic responses to firing activity

Here we have shown that bicuculline method of network disinhibition is a good stimulation paradigm with which to study energy use and energy production during network activity. Bicuculline-induced firing activity results in increased oxygen consumption, indicative of increased oxidative phosphorylation. This is likely to be mediated substantially by the cytoplasmic Ca^2+^-dependent activation of the Aralar component of the malate–aspartate shuttle (MAS) which functions to deliver reducing equivalents into the mitochondria (to reduce mitochondrial NAD + to NADH) as well as promoting generation of pyruvate [32,63]. Additionally, increased flux through the glycolytic pathway is suggested by the fact that the cellular NADH:NAD ratio (as measured by Peredox) increases sharply. Moreover, increased mitochondrial NADH levels in response to burst activity are clearly visible in the NADH autofluorescence imaging experiments. This increase is potentially due to both increased supply of electrons via the MAS, as well as activation/dephosphorylation of pyruvate dehydrogenase (Fig. 3G) and activation of Ca^2+^ dependent enzymes within the TCA cycle [27,34].

The energy cost of synaptic transmission and action potential firing is dealt with in excellent reviews elsewhere [50]. However, a key conclusion of previous studies is the very high energy cost associated with glutamate receptor activation (and recovery therefrom), which represents around 50% of all energy associated with synaptic transmission and AP firing [44]. As such, dendrites are likely to be associated with particularly strong ATP consumption during network activity, potentially explaining the higher rate of ATP loss in this region, compared to the soma (Fig. 4F, G). Of course though, there may also be differences in the ability of dendritic mitochondria to boost energy production, compared to those at the soma, for example due to a more limited substrate supply. The activity-dependent increase in NADH within dendrites was observed to be lower than at the soma (Fig. 4A), hinting at such a scenario. Alternatively, the temporal Ca^2+^ dynamics within dendrites may be less efficient at promoting the type of adaptive processes described in the previous paragraph. During burst activity, both cytoplasmic and mitochondrial Ca^2+^ levels are lower than at the soma (Fig. 4C, E), at least in part due to more rapid clearance of Ca^2+^ from the dendrites in between bursts (see Fig. 4B and [48]). As a result, the Ca^2+^-dependent activation of Aralar or of matrix dehydrogenases could potentially be weaker within dendrites.

## 4 Concluding remarks

The development of genetically encoded probes by a number of laboratories for a variety of second messengers and metabolites is enabling changes in these to be tracked with greater accuracy and spatio-temporal resolution. The considerable differences in the responses of dendrites and soma to a variety of challenges described in this study have been illuminated thanks to these newly developed probes as well as more established ones. Collectively they point to dendrites being particularly vulnerable to both oxidative stress as well as energy deficits. Since many neurodegenerative diseases and disease-causing agents are associated with excessive reactive species production and metabolic/bioenergetic perturbations, this increased vulnerability is potentially a contributing factor to early dendritic changes in a variety of neurodegenerative diseases.

## 5 Methods

### 5.1 Cell culture

Cortical neurons were cultured from E17.5 CD1 mouse embryos, essentially as previously described [2,81], at a density of between 9–13 × 10^4^ neurons per cm^2^. Cultures were prepared in Neurobasal growth medium plus 1% rat serum (Harlan Laboratories), B27 (Life Technologies Ltd), 1 mM glutamine and 1x antibiotic/antimycotic (Life Technologies Ltd). To prevent excessive astrocyte proliferation in neuronal cultures, the anti-mitotic drug cytosine β-d-arabino-furanoside hydrochloride (1.2 mM) was applied on DIV4. Cultures were utilised as indicated between DIV9–11, and were fed with the above described appropriate growth medium on DIV4. Prior to transfection cells were removed from growth medium and washed and placed in a minimal defined medium [79] containing 10% Minimum Essential Media (MEM, Life Technologies Ltd) and 90% salt–glucose–glycine (SGG) medium [6], which is comprised of 114 mM NaCl, 0.219% NaHCO_3_, 5.292 mM KCl, 1 mM MgCl_2_, 2 mM CaCl_2_, 10 mM HEPES, 1 mM glycine, 30 mM glucose, 0.5 mM sodium pyruvate, and 0.1% Phenol Red; osmolarity 325 mosm/l for at least 3 h.

### 5.2 General imaging parameters

Imaging was performed at 37 °C in ACSF (in mM): NaCl (150), KCl (3), HEPES (10), glycine (0.1), CaCl_2_ (2), MgCl_2_ (1), and glucose (10), pH 7.4 (this was used in all imaging experiments). Images were captured using a DFC350 FX digital camera as part of a Leica AF6000 LX imaging system. Dendritic ROIs chosen were greater than 2 cell bodies away from the soma and at least 5x narrower.

### 5.3 Peredox calibration, imaging and data analysis

Peredox is a genetically encoded, circularly permuted fluorescent reporter of the NADH/NAD + ratio. The probe was excited at 387 ± 5 nm and 575 ± 12 nm and emission collected at 530 ± 20 nm and 628 ± 14 nm. Peredox can be calibrated by replacing the glucose in the aCSF with lactate and/or pyruvate (both Sigma), which are the products and substrates of the lactate dehydrogenase (LDH). Lactate will cause LDH to produce NADH (hence increase Peredox fluorescence), while adding pyruvate will have the opposite effect. Different lactate:pyruvate ratios were applied to neurons transfected with Peredox in order to obtain a relationship between the lactate:pyruvate ratio and the percentage of maximum Peredox fluorescence, with lactate alone set to 100%. Lactate:pyruvate ratios were converted into NADH/NAD + levels by using the LDH equilibrium constant (k), with k = (pyruvate ∗ NADH) / (lactate ∗ NAD +) and k = 1.11 ∗ 10^− 4^. This allows to convert the % of maximum fluorescence (achieved by washing on lactate only) into NADH/NAD + ratios. To calibrate single experiments, at the end of each recording, aCSF containing lactate (10 mM) was applied to obtain the maximum fluorescence of Peredox and fluorescence was converted into NADH/NAD + ratios. All measured values were normalised to the signal obtained from mCherry, which is tagged to the Peredox protein. Images were taken every 20 s.

### 5.4 GCaMP2 Ca^2+^ imaging

Neurons were transfected with GCaMP2-encoding vectors (targeted to various locations), the fluorescence signal of which was detected using a standard GFP filter set (ex 480 ± 20; em 527 ± 15). Changes in Ca^2+^ were expressed as (F − F_min_) / (F_max_ − F) according to the equation [Ca^2+^] = Kd*(F − F_min_) / (F_max_− F). F_max_ was obtained when cells were treated with the cell-permeable Ca^2+^ ionophore ionomycin which both inserts into the plasma membrane and passes into the cell, inserting into mitochondrial membranes [75], leading to saturation of the indicator when in regular medium (2 mM Ca^2+^). F_min_ was obtained under the same conditions except in zero Ca^2+^ medium. The linear relationship between [Ca^2+^] and (F − F_min_) / (F_max_ − F) has been previously confirmed by calibrating the indicator as expressed in neurons, exposing them to ionomycin in the presence of sequentially different solutions of precise [Ca^2+^], obtained by mixing K_2_EGTA and CaEGTA solutions (Calcium Calibration Buffer Kit, Invitrogen) at different ratios [82].

### 5.5 Mitochondrial membrane potential imaging, and data analysis

Mitochondrial membrane potential was analysed as described [49,57] using Rh123 (Molecular Probes). Briefly, neurons were loaded with Rh123 (10 μg/ml or 26 μM) in SGG medium for 10 min followed by extensive washing with SGG. Rh123 partitions into the polarised mitochondria where it self-quenches at the concentration used. When the mitochondria depolarise, Rh123 leaks out of the mitochondria into the cytoplasm where it dequenches and fluoresces strongly. Maximum Rh123 signal (ex 480 ± 20; em 527 ± 15) was obtained by completely eliminating the mitochondrial potential by exposing the neurons to the mitochondrial uncoupler FCCP (5 μM; Sigma). The NMDA-induced change in Rh123 fluorescence was monitored in a number of cell bodies, plus in adjacent dendritic regions, normalised to the maximal (FCCP-induced) signal within that region of interest. Background fluorescence (the signal obtained in an area devoid of cells) was subtracted from images prior to any analysis.

### 5.6 NAD(P)H autofluorescence imaging

To measure NAD(P)H autofluorescence, cells were excited at 387 ± 5 nm and emitted light was collected at 447 ± 30 nm. Images were acquired every 20 s. 10–20 somas and dendritic areas were selected per recording. The drop in NAD(P)H autofluorescence was recorded until levels plateaued (~ 300 s post-NMDA). For experiments where BiC/4-AP was applied to the cells, NAD(P)H autofluorescence was measured for 10–16 min.

### 5.7 ATP (AT1.03) imaging and data analysis

AT1.03 is a YFP/CFP based FRET probe reporting ATP levels. Transfected cells were excited at 427 ± 5 nm and CFP and YFP/FRET emission was collected at 472 ± 15 nm and 542 ± 13 nm, respectively. Images were acquired every 5 s. To calibrate AT1.03, the mitochondrial uncoupler FCCP (10 μM) was applied, which deprives cells of ATP and gives the minimum AT1.03 fluorescence. After background subtraction, the YFP/CFP ratio was calculated for each region of interest. The YFP/CFP ratio obtained with FCCP was subtracted from the raw YFP/CFP ratio. The drop in ATP following the application of NMDA or BiC/4-AP was described as the change in the YFP/CFP ratio from the baseline.

### 5.8 GSH redox potential imaging (Grx1-roGFP2) and data analysis

Grx1-roGFP2 is a genetically encoded reporter of the glutathione redox potential. It was excited at 387 ± 5 and 494 ± 10 nm and emission collected at 530 ± 10. Images were acquired every 20 s. The 387/494 ratio was calculated after background subtraction. In order to investigate perturbations of the redox potential, 10 μM H_2_O_2_ (Sigma) was added to Grx1-roGFP2 transfected cells, which causes a non-saturating increase in the 387/494 ratio (peak responses were calculated). In order to calibrate the sensor, 100 μM H_2_O_2_ was applied to the cells, at which Grx1-roGFP2 reaches saturation.

### 5.9 Electrophysiological recording and analysis

Coverslips containing cortical DIV9–10 neurons were transferred to a recording chamber perfused (at a flow rate of 3–5 ml/min) with an external recording solution composed of (in mM): 150 NaCl, 2.8 KCl, 10 HEPES, 2 CaCl2, 1 MgCl2, 10 glucose, and 0.1 glycine, pH 7.3 (320–330 mOsm). Patch-pipettes were made from thick-walled borosilicate glass (Harvard Apparatus, Kent, UK), with a tip resistance ranging between 4–8 MΩ, and filled with a K-gluconate-based internal solution containing (in mM): K-gluconate 141, NaCl 2.5, HEPES 10, and EGTA 11; pH 7.3 with KOH. Recordings were at room temperature (21 ± 2 °C) using a Multiclamp 200B amplifier (Molecular Devices, Union City, CA). Both before and after current-clamp recordings, neurons were voltage-clamped at − 60 mV, and recordings were rejected if the holding current was greater than − 100 pA or if the series resistance drifted by more than 20% of its initial value (< 30 MΩ). Synaptic events were filtered at 5 kHz and digitized online at 20 kHz via a BNC-2090A/PCI-6251 DAQ board interface (National Instruments, Austin, TX, USA) and analysed using WinEDR 3.2 software (Dr John Dempster, University of Strathclyde, Glasgow, UK).

### 5.10 Transfection and plasmids

Neuronal transfections were carried out using Lipofectamine 2000 (2.33 μl/well, 1 μg/ml, Life Technologies Ltd) on DIV8 neurons. Plasmids used have been previously described: pCAGGS–GCaMP2 was a gift from Karel Svoboda [67]; GCaMP2-mt was a gift from Xianhua Wang [15]; Grx1-roGFP2 was a gift from Tobias Dick [42]; and Peredox-NLS was a gift from Gary Yellen [51].

### 5.11 Measurement of oxygen consumption rate

Oxygen consumption rate (OCR) was measured using the Seahorse XF24 extracellular flux analyser (Seahorse Bioscience). Cells were plated on poly-d-lysine and laminin coated XF24 plates. One hour before the experiment, the medium was changed to XF assay medium (modified DMEM), which does not include sodium bicarbonate, supplemented with 10 mM glucose and pH adjusted to 7.4. The following drugs were added sequentially: BiC/4-AP (50/250 μM), oligomycin (1 μM), FCCP (0.125 μM) and antimycin A/rotenone (both 2 μM; except for BiC/4-AP, all from Seahorse Bioscience). Oligomycin will inhibit oxygen consumption that is due to ATP production, while FCCP will uncouple the mitochondria and thus increase mitochondrial respiration, showing the cell's maximal respiratory capacity. After addition of antimycin A and rotenone, which will fully inhibit mitochondrial respiration, only non-mitochondrial oxygen consumption will be measured.

### 5.12 Western blotting

Gel electrophoresis and western blotting were performed using the Xcell Surelock system (Invitrogen) using precast gradient gels (4–20%) as described [68]. The following antibodies were used: Anti-PDH-E1α (pSer293), Merck Millipore 1:10,000; and Anti-PDH-E1α, Abcam, 1:10,000. For visualisation of Western blots, HRP-based secondary antibodies were used followed by chemiluminescent detection on Kodak X-Omat film. Western blots were analysed by digitally scanning the blots, followed by densitometric analysis (ImageJ). For figure preparation of example western blots, linear adjustment of brightness/contrast was applied (Photoshop) equally across the image.

### 5.13 Statistical analysis

Paired Student t-tests were used to compare non-independent data pairs, while Student t-tests were utilised when the two groups were not related. For studies employing multiple testing, we used a one-or two-way ANOVA followed by Bonferroni's post-hoc test. In most cases dendritic and somatic responses within the same cell were being compared, meaning that a repeated measure ANOVA was appropriate. For all tests significance was set at *p < 0.05. Error bars represent standard error of the mean.
